# Swiss paediatric dentists’ preferences and experience on the use of articaine and other local/topical anaesthetics

**DOI:** 10.1007/s40368-023-00852-9

**Published:** 2023-11-27

**Authors:** L. Baumgartner, S. N. Papageorgiou, H. van Waes, B. Hamza

**Affiliations:** Clinic of Orthodontics and Pediatric Dentistry, Center for Dental Medicine, University of Zurich, Plattenstrasse 11, 8032 Zurich, Switzerland

**Keywords:** Paediatric dentistry, Local anaesthetics, Articaine, Survey

## Abstract

**Purpose:**

This study was conducted to explore the preference and experience of paediatric dentists based in Switzerland regarding the use of articaine and other local and topical anaesthesia.

**Methods:**

An 18-question survey was developed, piloted, and distributed to the members of the Swiss association of paediatric dentistry (*n* = 460). The following information were collected: most used local anaesthetic in different age groups, time needed to inject a full ampule, frequency of observed local and systemic side effects, application of topical anaesthetic prior to injection, time waited between application and the injection, and perceived effectiveness of topical anaesthetic. The dentists’ responses were analysed with logistic regressions reporting odds ratios (OR) and 95% confidence intervals (CI) at 5%.

**Results:**

The response rate was 37% (*n* = 168) out of the 460 questionnaires sent, with the responders being predominantly female (67%) and 47-year-old on average. More than 80% of the dentists used articaine in all age groups. 45% of responders took longer than 60 s to inject a full ampule. Local and systemic side-effects were observed by 82% and 28% of respondents respectively, although the nature and the significance of those were not detailed due to the anonymous nature of the questionnaire. Significantly less local adverse effects were seen for older children (*p* = 0.04) and among dentists with more years of experience (*p* = 0.01). Most responders applied topical anaesthetic and half of them waited longer than 60 s before injection.

**Conclusions:**

Articaine is a widely used local anaesthetic by the studied group of Swiss paediatric dentists regardless of patient’s age. The use of topical anaesthetic before injection is a common practice with good perceived effectiveness.

**Supplementary Information:**

The online version contains supplementary material available at 10.1007/s40368-023-00852-9.

## Introduction

Effective pain management is one of the most important factors to facilitate the patient’s cooperation during dental treatment and is achieved by administering topical and/or local anaesthetics (Campbell et al. 2018). Local anaesthetics interrupt the line of communication between the source of pain and the central nervous system by intervening in the physiology of nerve stimulation (Ogle and Mahjoubi 2012). As excessive fear and anxiety—which are often encountered in paediatric patients—can increase pain perception during injection of local anaesthetics and even reduce the efficacy of anaesthesia (McGrath and McAlpine 1993), it is important to carefully choose an anaesthetic that minimises both the number of injections as well as the amount of anaesthetic needed (Monteiro et al. 2020).

Cocaine is the first chemically isolated local anaesthetic and is known for its short anaesthetic duration, its neuro- and cardio-toxicity, and its allergic response (Tobe et al. 2018). Since then, clinical trials in anaesthesia have long strived to counteract these side effects. Modern local anaesthetics have the molecular structure of an aromatic compound and an amino group, which are connected via an intermediate chain. Esters and amides, the main groups of local anaesthetics, differ in the type of intermediate chain they possess. Older anaesthetics such as procaine and tetracaine are esters, more recent ones like lidocaine, mepivacaine and articaine are amides (Mundiya and Woodbine 2022).

Since its introduction in the 1940s, lidocaine has been established as the most used local anaesthetic and has been labelled as the “gold standard” in dentistry (Mundiya and Woodbine 2022; Saraf et al. 2016). Nevertheless, the search for even a better anaesthetic went on (Taneja et al. 2020). The search resulted in the development of mepivacaine in the early 1960s, which was reported to increase the success rate of local anaesthesia and pain control during the injection with the same toxicity compared to lidocaine at the same concentration of adrenaline (Su et al. 2014). Articaine was developed in the 1970s and was also considered a better alternative to lidocaine. This proclaimed superiority for articaine was attributed to achieving significantly more successful anaesthesia results than lidocaine for infiltration and mandibular block anaesthesia, whilst being considerably less toxic (Brandt et al. 2011; Leith et al. 2012; Larocca de Geus et al. 2020). Although articaine is a local anaesthetic of the amide type, it differs essentially from the other amides due to the replacement of the benzene ring through a thiophene ring that increases its lipid-solubility and potency (Leith et al. 2012). Another benefit of articaine is its rapid metabolisation into an inactive form both in the plasma as well as in tissues, owing to the presence of the hydrolysing ester group (Moore and Hersh 2010; Decloux and Ouanounou 2020). Whilst the superiority of articaine over lidocaine in pain management, based on the subjective feelings of children, was reported in some reviews (Taneja et al. 2020), other reviews reported such findings to lack sufficient evidence (Klingberg et al. 2017).

The use of articaine in young children has often been a matter of hesitation among paediatric dentists (Leith et al. 2012). This is largely due to the manufacturers not recommending its use in children younger than 4 years old. Nevertheless, articaine use in this age group has been reported in the literature. Wright et al. (1989) reviewed the records of 211 children under 4 years of age who received articaine with or without sedation and reported no adverse effects even when the administered dosage exceeded the recommended one. Still, the systematic review and meta-analysis of Katyal (2010) did not recommend the use of articaine in children under 4 years of age due to the lack of supporting data.

Earlier analyses of the preferred local anaesthetic used by paediatric dentists were conducted in the United Kingdom and the United States. Both populations reported to prefer using lidocaine over articaine (Ezzeldin et al. 2020; Brickhouse et al. 2008). On the other hand, a policy document of the European Academy of Paediatric Dentistry (EAPD) stated that articaine was the most preferred injectable local anaesthetic in several European countries (Kühnisch et al. 2017). To the best of the author’s knowledge, no such analysis has been conducted in Switzerland. Therefore, the aim of this study was to explore the preference and experience of paediatric dentists based in Switzerland regarding the use of articaine and other local and topical anaesthesia in children.

## Methods

Members of the Swiss association of paediatric dentistry (*n* = 460) were invited by email to participate in an online survey created in Google Docs, which was based on previously existing similar studies (Kohli et al. 2001; Ezzeldin et al. 2020). The survey questions were translated into German and French by a native speaker of both languages, according to the language spoken in each canton. The need of an ethical approval for the conduction of this study was waived by Zurich cantonal ethics committee (BASEC-Nr. Req-2022-00960). An online search in known dental-material providers in Switzerland was conducted to define which local anaesthetics were at all available for purchase at the time of the study. Before the online invitations were sent, the survey was piloted among a small group of paediatric dentists in the University of Zurich (*n* = 20) and modified according to the received feedback. The emails were sent in the beginning of January 2023, a reminder was sent in the middle of February 2023 and the survey was open for participation until the middle of March 2023. The sent invitations explained the anonymous and the voluntary nature of the survey and included links for either the German or the French version of the survey. This study is reported in accordance with the Strengthening the Reporting of Observational Studies in Epidemiology (STROBE) statement (von Elm et al. 2008).

The survey consisted of 18 questions and started by collecting the demographic information of the dentist (age, gender, year and university of graduation, and the approximate percentage of children they treat in their praxis in relation to their whole patient population). This was followed by questions regarding the preferred local anaesthetic based on different weights and ages, the main factors considered when deciding the used doses, and the approximate time needed to inject a full ampule of the local anaesthetic. The dentists were then asked about their preference and experience on applying topical anaesthetics prior to needle injection. The final questions were dedicated to the frequency of observed local and/or systematic side effects of the used local anaesthetics and the preferred method of administering them. Table 1 shows the survey questions and the paediatric dentists’ answers.

**Table 1:** The survey questions and the dentists’ answers

Question	Answers
Please enter your age (*n* = 168)	
Median (IQR)	47.0 (37.0 to 55.5)
Range	24.0 to 75.0
What is your gender? (*n* = 168)	
Female–*n* (%)	112 (66.7%)
Male–*n* (%)	55 (32.7%)
Other–*n* (%)	1 (0.6%)
Which university did you graduate from? (*n* = 164)	
Basel–*n* (%)	28 (17.1%)
Bern–*n* (%)	35 (21.3%)
Geneva–*n* (%)	11 (6.7%)
Zurich—*n* (%)	68 (41.5%)
Other–*n* (%)	22 (13.4%)
Which year did you graduate in? (*n* = 168)	
1970s–*n* (%)	1 (0.6%)
1980s–*n* (%)	27 (16.1%)
1990s–*n* (%)	41 (24.4%)
2000s–*n* (%)	48 (28.6%)
2010s–*n* (%)	41 (24.4%)
2020s–*n* (%)	10 (6.0%)
Years of experience (*n* = 168)	
Median (IQR)	19.0 (11.5, 30.0)
Range	1.0–50.0
What is the percentage of children (0–12 years) amongst all your patients? (*n* = 168)	
0–50%–*n* (%)	78 (46%)
51–90%–*n* (%)	33 (19.6%)
> 90%–*n* (%)	57 (33.9%)
What type of local anaesthetic do you use most often in children younger than 4 years (up to 20 kg)? (*n* = 168)	
Lidocaine with/without adrenaline–*n* (%)	6 (3.6%)
Articaine with adrenaline (1:100′000)–*n* (%)	26 (15.5%)
Articaine with adrenaline (1:200′000)–*n* (%)	80 (47.6%)
Articaine with adrenaline (1:400′000)–*n* (%)	31 (18.5%)
Mepivacaine 3%–*n* (%)	5 (3.0%)
Not applicable–*n* (%)	18 (10.7%)
Other–*n* (%)	2 (1.2%)
What type of local anaesthetic do you use most often in children between 4 and 12 years (up to 45 kg)? (*n* = 168)	
Lidocaine with/without adrenaline–*n* (%)	6 (3.6%)
Articaine with adrenaline (1:100′000)–*n *(%)	39 (23.2%)
Articaine with adrenaline (1:200′000)–*n* (%)	92 (54.8%)
Articaine with adrenaline (1:400′000)–*n* (%)	27 (16.1%)
Mepivacaine 3%–*n* (%)	2 (1.2%)
Not applicable–*n* (%)	1 (0.6%)
Other–*n *(%)	1 (0.6%)
What type of local anaesthetic do you use most often in adolescents older than 12 years (> 45 kg)? (*n* = 168)	
Lidocaine with/without adrenaline–*n* (%)	5 (3.0%)
Articaine with adrenaline (1:100′000)–*n* (%)	44 (26.2%)
Articaine with adrenaline (1:200′000)–*n* (%)	95 (56.6%)
Articaine with adrenaline (1:400′000)–*n* (%)	20 (11.9%)
Mepivacaine 3%–*n* (%)	2 (1.2%)
Not applicable–*n* (%)	1 (0.6%)
Other–*n* (%)	1 (0.6%)
What is the most important factor that you consider when you decide on the dosage of the local anaesthetic? (*n* = 168)	
Age only–*n* (%)	4 (2.3%)
Weight only–*n* (%)	86 (51.1%)
Age and weight–*n* (%)	69 (41.0%)
Other–*n* (%)	13 (7.7%)
How long do you take to inject a full ampule? (*n* = 168)	
< 10 s–*n* (%)	1 (0.6%)
10–30 s–*n* (%)	19 (11.3%)
30–60 s–*n* (%)	72 (42.9%)
> 60 s–*n* (%)	76 (45.2%)
How often do you observe local side effects after injecting local anaesthetics (e.g., Lip bite)? (*n* = 168)	
Never–*n* (%)	30 (17.9%)
≤ 1/year–*n* (%)	83 (49.4%)
2–5/year–*n* (%)	47 (28.0%)
> 5/year–*n* (%)	8 (4.8%)
How often do you observe systemic side effects after injecting local anaesthetics? (*n* = 168)	
Never–*n* (%)	121 (72.0%)
≤ 1/year–*n* (%)	35 (20.8%)
2–5/year–*n* (%)	12 (7.1%)
> 5/year–*n* (%)	0 (0%)
Do you use any topical anaesthetic before an injection? (*n* = 168)	
Yes–*n* (%)	151 (89.9%)
No–*n* (%)	17 (10.1%)
How often do you use a topical anaesthetic before an injection in children (younger than 12)? (*n* = 151)	
Rarely–*n* (%)	0 (0%)
Sometimes–*n* (%)	10 (6.6%)
Always–*n* (%)	141 (93.4%)
How often do you use a topical anaesthetic before an injection in adolescents (older than 12)? (*n* = 151)	
Rarely–*n* (%)	24 (15.9%)
Sometimes–*n* (%)	46 (30.5%)
Always- *n* (%)	81 (53.6%)
How long do you wait after applying a topical anaesthetic before injecting? (*n* = 151)	
< 30 s–*n* (%)	13 (8.6%)
30–60 s–*n* (%)	65 (43.1%)
> 60 s–*n* (%)	73 (48.3%)
How do you rate the effectiveness of topical anaesthetics in reducing injecting pain? (*n* = 151)	
Ineffective–*n* (%)	0 (0.0%)
Little effective–*n* (%)	15 (9.9%)
Effective–*n* (%)	91 (60.3%)
Very effective–*n* (%)	45 (29.8%)
You are treating a 6-year-old child with deep caries in a primary second lower molar. Which type of anaesthesia do you choose? (*n* = 168)	
Nerve block anaesthesia–*n* (%)	36 (21.4%)
Infiltration anaesthesia–*n* (%)	132 (78.6%)

*IQR* Interquartile range

### Statistical analysis

Initially, descriptive statistics were calculated, including absolute/relative frequencies for categorical variables and medians with interquartile ranges for continuous variables (after appropriate check with the Shapiro–Wilk test). Participating dentists were categorised into three groups according to the percentage of children they treat in relation to the whole patient population (up to 50%, between 50 and 90%, and between 90 and 100%). The influence of this categorisation on the survey’s answers was assessed with logistic regressions and expressed as odds ratios (OR) and 95% confidence intervals (CI). All analyses were performed in Stata SE 13.0 (StataCorp, College Station, TX, USA) with two-sided alpha = 5%, and an open dataset (https://doi.org/10.5281/zenodo.8373961).

## Results

One hundred and sixty-eight paediatric dentists (out of the 460 sent invitations) completed the online survey, to a response/completion rate of 37%. Of those, 112 (67%) were female. The median age of participants was 47 years (IQR 37 to 55 years) and the median years of experience was 19 years (IQR 12 to 30 years), whilst 87% graduated from a Swiss university. Seventy-eight dentists (46%) reported that children accounted for up to 50% of their whole clientele, 33 (19%) more than 50%, and 57 (34%) more than 90%.

Regarding the used local anaesthetic in children younger than 4 years of age, most of the dentists (*n* = 137; 82%) reported using articaine with different concentrations of adrenalin, whilst the percentage of patients being children did not seem to influence this (*p* = 0.71; Table 2). Articaine was still the most used local anaesthetics in 4- to 12-year-old children (*n* = 158; 94%), and also in adolescents older than 12 years (*n* = 159; 95%). Body weight was the most important factor considered when calculating the maximum dose of the local anaesthetic (*n* = 86; 51%) followed by the combination of age and body weight (*n* = 69; 41%). Sixty-five dentists (43%) reported that they would take 30 to 60 s to inject a full ampule of local anaesthetic, whilst 73 of them (45%) reported they would take more than 60 s to do so. The greater the percentage of patients treated being children, the longer the dentist took to inject a full ampule of the local anaesthetic (*p* < 0.001; Table 3).

**Table 2:** Factors associated with using anaesthetic without articaine for children aged 0–4 years

Factor	Level	OR (95% CI)	*p*
Part children	Up to 50%	Reference	0.71*
	51–90%	0.50 (0.06, 4.46)	
	> 90%	1.24 (0.34, 4.52)	

*CI* Confidence interval, *OR* odds ratio

*Overall Wald-type test

**Table 3:** Differences of time needed to inject a whole ampule according to part of patients being children

Part children	< 30 s	30–60 s	> 60 s	*p* (exact)
≤ 50%	13 (16.7%)	44 (56.4%)	21 (26.9%)	< 0.001
51–90%	2 (6.1%)	15 (45.5%)	16 (48.5%)	
> 90%	5 (8.8%)	13 (22.8%)	39 (68.4%)	

Regarding observed side effects, 30 dentists (18%) reported never observing local side effects and 83 dentists (49%) reported observing them once or less annually. Dentists with more than 90% patients being children were less likely to observe a local side effect (*p* < 0.001; Table 4). Years of experience seemed to play a protective role in this aspect, as dentists with more years of experience reported seeing local side effects less often (*p* = 0.01; Table 4). One hundred twenty-one dentists (72%) reported never observing systemic side effects and 35 dentists (21%) reported observing them once or less annually. Again, dentists with more than 90% patients being children were less likely to observe such side effects (*p* < 0.001), but years of experience didn’t seem to play a role (*p* = 0.6; Table 5).

**Table 4:** Factors associated with seeing local adverse effects more than once/year

Factor	Level	OR (95% CI)	*p*
Age	Per year	0.97 (0.94, 1.00)	0.04
Sex	Female	Reference	
	Male	0.52 (0.25, 1.07)	0.08
Swiss university	Basel	Reference	0.34*
	Bern	1.88 (0.65, 5.40)	
	Geneva	3.00 (0.71, 12.69)	
	Zurich	1.20 (0.46, 3.14)	
Country	Switzerland	Reference	
	Other	0.32 (0.11, 0.99)	0.04
Part children	Up to 50%	Reference	< 0.001*
	51–90%	0.59 (0.25, 1.41)	
	> 90%	0.10 (0.03, 0.31)	
Years experience	Per year	0.96 (0.94, 0.99)	0.01
Topical anaesthetic	No	Reference	
	Yes	1.66 (0.51, 5.34)	0.40

*CI* Confidence interval, *OR* odds ratio

*Overall Wald-type test

**Table 5:** Factors associated with seeing systemic adverse effects at least once/year

Factor	Level	OR (95% CI)	*p*
Age	Per year	0.99 (0.96, 1.02)	0.37
Sex	Female	Reference	
	Male	1.22 (0.60, 2.48)	0.58
Swiss university	Basel	Reference	0.74*
	Bern	0.72 (0.25, 2.09)	
	Geneva	0.40 (0.07, 2.23)	
	Zurich	0.70 (0.27, 1.78)	
Country	Switzerland	Reference	
	Other	0.74 (0.28, 1.97)	0.55
Part children	Up to 50%	Reference	< 0.001
	51–90%	0.59 (0.25, 1.41)	
	> 90%	0.10 (0.03, 0.31)	
Years experience	Per year	0.99 (0.96, 1.02)	0.68
Surface anaesthetic	No	Reference	
	Yes	0.92 (0.31, 2.78)	0.89

*CI* Confidence interval, *OR* odds ratio

*Overall Wald-type test

One hundred fifty-one dentists (90%) reported applying a topical anaesthetic prior to an injection with its use not being formally associated with the percentage of child patients (*p* = 0.05; Supplementary file 1). Among those, 141 (93%) reported always applying a topical anaesthetic in children younger than 12 years of age. This number dropped to 81 (54%) in adolescents older than 12 years of age. In adolescents, an increasing percentage of treated children was associated with increased odds that the dentist applied a topical anaesthetic (*p* < 0.001; Supplementary file 2). However, this association was less evident in children younger than 12 years of age (*p* = 0.05; Supplementary file 3). In other words, both groups of dentists (those with up to 50% patients being children, and those with up to 90% patients being children) tended to apply a topical anaesthetic before the injection more frequently in children younger than 12 years than in adolescents older than 12 years. Sixty-five dentists reported to wait 30 to 60 s between the application of the topical anaesthetic and the introduction of the injection, whilst 73 dentists (48%) reported to wait for more than 60 s. The percentage of patients being children did not seem to influence this aspect (*p* = 0.39; Supplementary file 4). Ninety-one dentists (60%) believed topical anaesthetic to be effective in reducing pain during the injection, and 45 dentists (30%) believed it to be even very effective in doing so. The time waited between the application of the topical anaesthetic and the introduction of the injection seemed to influence the perceived effectiveness of the topical anaesthetic (i.e., the longer the time, the more effective the topical anaesthetic was perceived) (*p* = 0.01; Supplementary file 5).

## Discussion

Administering local anaesthetics is one of the most common practices in a dental clinic. The achieved pain elimination plays especially an important role when treating children. Articaine has been considered superior to lidocaine due to its increased lipid-solubility and potency. However, its use in children younger than 4 years of age is still not recommended by the manufacturers. This study investigated the experience of paediatric dentists based in Switzerland regarding the use of articaine and other local/topical anaesthetics.

Articaine was—by far—the most used local anaesthetic by more than 80% of the participating Swiss paediatric dentists in all age groups (0–4, 4–12, and older than 12), compared to 3% who chose lidocaine. The fact that participating Swiss paediatric dentists widely use articaine in children younger than 4 years of age is especially interesting, as articaine is contraindicated in this group of children in Switzerland (Arzneimittel-Kompendium 2023). In Austria, however, a generic of articaine (Ultracain Dental forte) is approved for children older than one year. Anecdotally, two other generics of articaine (Ubistesin forte and Septanest mit Epinephrin) with the same concentrations of articaine and adrenaline as Ultracain Dental forte are only recommended in children older than 4 years of age due to the lack of data for younger children (Medizinmarktaufsicht 2023). This discrepancy in the age approval and labelling (“not recommended”, “lack of sufficient data”, “contraindicated”) between similar products in the same and neighbouring countries might be caused by bureaucratic formalities, and might be in part responsible for the fact that Swiss paediatric dentists use articaine in an—officially—contraindicated age group. This unsatisfactory aspect was also discussed in the latest policy document on best clinical practice for administering local anaesthesia in paediatric patients by the European Academy of Paediatric Dentistry (EAPD). The document has also drawn attention to other notable gaps in our current understanding in this particular area (e.g., dosage recommendations in relation to the dental treatment and evidence for the most effective and comfortable injection technique) (Kühnisch et al. 2017).Another reason of this wide use of articaine in younger children could be the lack of knowledge that a contraindication exists.

The here-reported wide use of articaine is in contrast with a couple of studies in the literature. In the United Kingdom, Ezzeldin et al. (2020) reported that only 19% of the members of the British society of paediatric dentistry chose articaine to be their first line anaesthetic, compared to 80% who chose lidocaine. However, articaine was still reported to be used by 62% of the paediatric dentists on daily and weekly basis. Of those using articaine, 87% reported refraining from using it in children younger than 4 years of age. In the United States, Brickhouse et al. (2008) reported that 10% of paediatric dentists chose articaine as their preferred anaesthetic in 2- to 3-year olds, compared to 82% who chose lidocaine. In 4- to 6-year-olds, the preference of articaine among paediatric dentists rose to 15%, compared to 78% who still preferred lidocaine. Alanazi et al. (2021) also reported that articaine was only used by 1.7% of Saudi dental practitioners when treating paediatric patients, compared to 92% who used lidocaine. Different marketing representations of each kind of anaesthetic and the kind of anaesthetic dentists used during their undergraduate studies might play a role in these contrasting findings (Ezzeldin et al. 2020). For instance, four out of six dentists who chose lidocaine as the most used anaesthetic in 0–4-year-olds in this study graduated from universities outside of Switzerland (United States, Germany, Greece).

Twenty-eight percent of participating Swiss paediatric dentists reported observing local side effects related to local anaesthetics 2 to 5 times annually, and 7% of them reported observing systemic side effects with the same frequency. Almost all of those reporting such frequency of observed side effects were articaine users in all age groups. However, and due to the considerably big difference between the numbers of articaine and lidocaine users in this study (137 vs. 6 in 0–4-year-old group, 158 vs. 6 in 4–12-year-old group, and 159 vs. 5 in > 12-year-old group), no statistically based conclusion can be made in favour of lidocaine (as the safer choice with less observed side effects). In fact, articaine might be considered–at least theoretically—systemically safer than lidocaine due to the presence of an ester side chain in its thiophene ring. The relative rapid inactivation of this chain in plasma results in the fact that articaine has a half-life of only 20 min compared to about 90 min in lidocaine. This shorter half-life is especially meaningful in lengthy appointments, where additional doses of anaesthetics might be necessary (Becker and Reed 2006). Among paediatric dentists in the UK, Ezzeldin et al. (2020) reported that the odds of side effects were greater when using lidocaine than when using articaine, considering the frequency of use. The reported side effects were prolonged paraesthesia and soft tissue trauma (84% in the lidocaine-user group vs. 15% in the articaine-user group). A higher proportion of participating Swiss paediatric dentists (approximately 70%) reported observing local side effects when using articaine on at least one occasion annually. In their systematic review, Klingberg et al. (2017) reported that no serious side or adverse effects were reported in the searched literature apart from soft tissue injuries such as lip or cheek biting, or pain related to injection site. Therefore, the relatively high percentage of participating Swiss paediatric dentists (28%) who reported observing systemic side effects was unexpected. Unfortunately, due to the anonymous nature of the questionnaire, further details regarding these observed systemic side effects could not be obtained. Ninety-three percent of participating Swiss paediatric dentists reported to always use a topical anaesthetic before injection. Similar result was reported by Kohli et al. (2001) where 89% of the paediatric dentists in the United States also reported to always use a topical anaesthetic. More percentage of Swiss paediatric dentists (48%) tended to wait for more than 60 s between topical anaesthetic and the injection than American peers (33%). Longer waiting times was found to be associated with more perceived effectiveness of topical anaesthetic in this study, which could be deemed logical as they would have more time to numb the injection site. This might explain the more percentage of Swiss paediatric dentists perceiving their application of topical anaesthetic as effective or very effective compared to the American peers (90% vs. 61%). Nevertheless, this explanation is rather speculative as many other factors could also play a role.

One potential limitation of this study is that the survey was distributed via email, which may have resulted in selection bias. Paediatric dentists who are more active or interested in research may be more likely to respond to the survey, potentially skewing the results. In Addition, self-reported data may not always be accurate or complete, as individuals may have different interpretations or memories of their experiences. Furthermore, the fact that the survey questions were close ended have limited the ability to collect more detailed and nuanced responses. At the same time, the attempt to fetch as much information as possible from a single survey might prolong the time necessary to answer all the questions and could affect the participation interest even more.

## Conclusion

Based on this cross-sectional study and within its limits, it could be stated that most participating Swiss paediatric dentists prefer the use of articaine in children younger and older than 4 years of age. The use of topical anaesthetic before injection is also a common practice with a good perceived effectiveness.

### Supplementary Information

Below is the link to the electronic supplementary material.

Supplementary file1 (DOCX 16 KB)

## Data Availability

Non-applicable.

## Data availability

Data used in this study can be accessed at (https://doi.org/10.5281/zenodo.8373961).

## References

[CR1] Alanazi FS, Alhazzaa MF, Alosaimi YM, Alajaji FA, Alanazi AS, Alassaf A, Almulhim B, Alghamdi SA, Mallineni SK (2021). Preference of dental practitioners toward the use of local and topical anesthetics for pediatric patients in Saudi Arabia: a cross-sectional survey. Children.

[CR2] Arzneimittel-Kompendium der Schweiz, https://compendium.ch/product/123475-rudocain-forte-inj-los/mpro#MPro7200. Accessed 01 May 2023.

[CR3] Becker DE, Reed KL (2006). Essentials of local anesthetic pharmacology. Anesth Prog.

[CR4] Brandt RG, Anderson PF, McDonald NJ, Sohn W, Peters MC (2011). The pulpal anesthetic efficacy of articaine versus lidocaine in dentistry: a meta-analysis. J Am Dent Assoc.

[CR5] Brickhouse TH, Unkel JH, Webb MD, Best AM, Hollowell RL (2008). Articaine use in children among dental practitioners. Pediatr Dent.

[CR6] Campbell RL, Shetty NS, Shetty KS, Pope HL, Campbell JR (2018). Pediatric dental surgery under general anesthesia: uncooperative children. Anesth Prog.

[CR7] Decloux D, Ouanounou A (2020). Local anaesthesia in dentistry: a review. Int Dent J.

[CR8] Ezzeldin M, Hanks G, Collard M (2020). United Kingdom pediatric dentistry specialist views on the administration of articaine in children. J Dent Anesth Pain Med.

[CR9] Katyal V (2010). The efficacy and safety of articaine versus lignocaine in dental treatments: a meta-analysis. J Dent.

[CR10] Klingberg G, Ridell K, Brogårdh-Roth S, Vall M, Berlin H (2017). Local analgesia in paediatric dentistry: a systematic review of techniques and pharmacologic agents. Eur Arch Paediatr Dent.

[CR11] Kohli K, Ngan P, Crout R, Linscott CC (2001). A survey of local and topical anesthesia use by pediatric dentists in the United States. Pediatr Dent.

[CR12] Kühnisch J, Daubländer M, Klingberg G, Dougall A, Spyridonos Loizides M, Stratigaki E, Amar JL, Anttonen V, Duggal M, Gizani S (2017). Best clinical practice guidance for local analgesia in paediatric dentistry: an EAPD policy document. Eur Arch Paediatr Dent.

[CR13] Larocca de Geus J, Nogueira da Costa JK, Wambier LM, Maran BM, Loguercio AD, Reis A (2020). Different anesthetics on the efficacy of inferior alveolar nerve block in patients with irreversible pulpitis: a network systematic review and meta-analysis. J Am Dent Assoc.

[CR14] Leith R, Lynch K, O'Connell AC (2012). Articaine use in children: a review. Eur Arch Paediatr Dent.

[CR15] McGrath PJ, McAlpine L (1993). Psychologic perspectives on pediatric pain. J Pediatr.

[CR16] Medizinmarktaufsicht. Bundesamt für Sicherheit im Gesundheitswesen, https://aspregister.basg.gv.at/aspregister/faces/aspregister.jspx. Accessed 01 May 2023.

[CR17] Monteiro J, Tanday A, Ashley PF, Parekh S, Alamri H (2020). Interventions for increasing acceptance of local anaesthetic in children and adolescents having dental treatment. Cochrane Database Syst Rev.

[CR18] Moore PA, Hersh EV (2010). Local anesthetics: pharmacology and toxicity. Dent Clin North Am.

[CR19] Mundiya J, Woodbine E (2022). Updates on topical and local anesthesia agents. Oral Maxillofac Surg Clin North Am.

[CR20] Ogle OE, Mahjoubi G (2012). Local anesthesia: agents, techniques, and complications. Dent Clin North Am.

[CR21] Saraf SP, Saraf PA, Kamatagi L, Hugar S, Tamgond S, Patil J (2016). A comparative evaluation of anesthetic efficacy of articaine 4% and lidocaine 2% with anterior middle superior alveolar nerve block and infraorbital nerve block: An in vivo study. J Conserv Dent.

[CR22] Su N, Liu Y, Yang X, Shi Z, Huang Y (2014). Efficacy and safety of mepivacaine compared with lidocaine in local anaesthesia in dentistry: a meta-analysis of randomised controlled trials. Int Dent J.

[CR23] Taneja S, Singh A, Jain A (2020). Anesthetic effectiveness of articaine and lidocaine in pediatric patients during dental procedures: a systematic review and meta-analysis. Pediatr Dent.

[CR24] Tobe M, Suto T, Saito S (2018). The history and progress of local anesthesia: multiple approaches to elongate the action. J Anesth.

[CR25] von Elm E, Altman DG, Egger M, Pocock SJ, Gøtzsche PC, Vandenbroucke JP, Initiative S (2008). The strengthening the reporting of observational studies in epidemiology (STROBE) statement: guidelines for reporting observational studies. J Clin Epidemiol.

[CR26] Wright GZ, Weinberger SJ, Friedman CS, Plotzke OB (1989). Use of articaine local anesthesia in children under 4 years of age–a retrospective report. Anesth Prog.

