# Supplementary material for: Swiss paediatric dentists’ preferences and experience on the use of articaine and other local/topical anaesthetics
Source: Eur Arch Paediatr Dent. 2023 Nov 27;25(1):49–56. doi: 10.1007/s40368-023-00852-9 (PMC10942887; doi:10.1007/s40368-023-00852-9)
Supplement: Supplementary file 1 — Supplementary file1 (DOCX 16 KB) [file 40368_2023_852_MOESM1_ESM.docx]

**Supplementary file 1.** Association of topical anaesthetic use by part of patients being children.

| **Factor** | **Level** | **OR (95% CI)** | **P** |
| --- | --- | --- | --- |
| Part children | Up to 50% | Reference | 0.05* |
|  | 51-90% | 2.00 (0.53, 7.55) |  |
|  | >90% | 11.2 (1.42, 88.32) |  |

CI, confidence interval; OR, odds ratio.

* overall Wald-type test

**Supplementary file 2.** Differences of topical anaesthetic use frequency for adolescents (aged >12 years) according to part of patients being children

| **Part children** | **Rarely** | **Sometimes** | **Always** | **P (exact)** |
| --- | --- | --- | --- | --- |
| ≤50% | 18 (27.7%) | 25 (38.5%) | 22 (33.9%) | <0.001 |
| 51-90% | 5 (16.7%) | 13 (43.3%) | 12 (40.0%) |  |
| >90% | 1 (1.8%) | 8 (14.3%) | 47 (83.9%) |  |

**Supplementary file 3.** Differences of topical anaesthetic use frequency for children (aged <12 years) according to part of patients being children

| **Part children** | **Sometimes** | **Always** | **P (exact)** |
| --- | --- | --- | --- |
| ≤50% | 8 (12.3%) | 57 (87.7%) | 0.05 |
| 51-90% | 1 (3.3%) | 29 (96.7%) |  |
| >90% | 1 (1.8%) | 55 (98.2%) |  |

**Supplementary file 4.** Differences of time waited from topical anaesthetic to injection according to part of patients being children

| **Part children** | **<30 sec** | **30-60 sec** | **>60 sec** | **P (exact)** |
| --- | --- | --- | --- | --- |
| ≤50% | 8 (12.3%) | 24 (36.9%) | 33 (50.8%) | 0.39 |
| 51-90% | 1 (3.3%) | 17 (56.7%) | 12 (40.0%) |  |
| >90% | 4 (7.1%) | 24 (42.9%) | 28 (50.0%) |  |

**Supplementary file 5.** Factors associated with judging topical anaesthetic as very effective.

| Factor | Level | OR (95% CI) | P |
| --- | --- | --- | --- |
| Time waited to injection | < 30 seconds | Reference | 0.01* |
|  | 30-60 seconds | 0.46 (0.12, 1.76) |  |
|  | >60 seconds | 1.57 (0.44, 5.57) |  |

CI, confidence interval; OR, odds ratio.

* overall Wald-type test
